# Tolerance to Lung Infection in TWIK2 K^+^ Efflux Mediated Macrophage Trained Immunity

**DOI:** 10.1101/2025.05.25.655979

**Published:** 2025-05-28

**Authors:** Josh Thompson, Yufan Li, Yuanling Song, Ki-Wook Kim, Asrar B. Malik, Jingsong Xu

**Affiliations:** 1 Department of Pharmacology and Regenerative Medicine, University of Illinois, Chicago, USA; 2 Center for Lung and Vascular Biology, University of Illinois, Chicago, USA; 3 Shanghai Key Laboratory of Lung Inflammation and Injury, Department of Pulmonary Medicine, Zhongshan Hospital, Fudan University, Shanghai, China

## Abstract

Alveolar macrophages (AMφ) are essential for innate immune function in the lungs. It is now apparent that macrophages can be trained to become better at attacking infections. Although trained immunity is thought to result from metabolic and epigenetic reprogramming, the underlying mechanisms remain unclear. Here, we report that AMφ can be trained by extracellular ATP, which is ubiquitously released during inflammation. ATP ligates the canonical Purinergic Receptor 2 subtype X7 receptor (P2X7) to mediate endosomal Two-pore domain Weak Inwardly rectifying K^+^ channel 2 (TWIK2) translocation into the plasma membrane (PM). This endows the cells to transit to a ‘ready’ state for microbial killing in two directions: first, K^+^ efflux via PM-TWIK2 induces NLRP3 inflammasome activation, which further activates metabolic pathways; second, upon bacterial phagocytosis, PM-TWIK2 internalizes into phagosome membrane with proper topological orientation, where TWIK2 mediates K^+^ influx into phagosomes to control pH and ionic strength favoring bacterial killing. Therefore, the enhanced association of TWIK2 in phagosomal and plasma membranes signaled by danger-associated molecular patterns (DAMPs), such as ATP, mediates trained immunity in AMφ and enhances the microbiocidal activity.

## Introduction

Immune responses are classically divided into innate immune responses, which react rapidly and nonspecifically upon encountering a pathogen, and adaptive immune responses, which are slower to develop, show a measured response that is specific and the result of acquiring immunological memory(Netea et al., 2016). Although innate immunity is considered a non-specific, short-lived phenomenon as opposed to adaptive immunity, which is long-lived and highly specific, recent studies have shown that innate immunity can display adaptive immunity characteristics after challenge with pathogens or their products(Netea et al., 2016). Trained immunity mediates protection against heterologous infections and is thought to be due to epigenetic and functional reprogramming of innate immune cells(Netea and Joosten, 2018). However, trained immunity also exhibits maladaptive effects in chronic inflammatory conditions such as cardiovascular diseases and autoinflammatory syndromes(Netea and Joosten, 2018).

AMφ are key effector cells of the innate immune response in the lungs, which not only play a key role in killing bacterial pathogens through processes such as phagocytosis or secretion of antimicrobial peptides, but also play an important role in the restoration of homeostasis(Allard et al., 2018). The efficiency of bacterial killing is an essential determinant of the ability to resolve lung inflammation and injury as seen in patients with acute lung injury (ALI) and acute respiratory distress syndrome (ARDS)(Sevransky et al., 2009). Being located at the interface of the airways and the environment, lung AMφ exhibit a distinctive cellular profile that tightly regulates their activation state to avoid excessive inflammation(Bain and MacDonald, 2022). Due to their unique properties, lung AMφ are important candidates for understanding trained immunity. Our current knowledge about innate immune memory responses of AMφ and the underlying mechanisms remains limited.

NLRP3 inflammasome is a key determinant of acute immune responses as seen in ALI/ARSD(Grailer et al., 2014; Sefik et al., 2022; Swanson et al., 2019). Activation of NLRP3 inflammasome complex is a multi-step process involving the assembly of key proteins and activation of caspase-1 which cleaves pro-Interleukin-1β (pro-IL-1β) to release the active form of this inflammatory cytokine(He et al., 2016; Swanson et al., 2019). However, little is known about the triggers that initiate the activation of the NLRP3 complex. An essential mechanism of NLRP3 assembly is the efflux of K^+^ at PM(Franchi et al., 2007; Munoz-Planillo et al., 2013; Petrilli et al., 2007) through the K^+^ channel TWIK2(Di et al., 2018; Enyedi and Czirjak, 2010). The efflux of K^+^ generates regions of low intracellular K^+^, which promote a conformational change of inactive NLRP3 to facilitate NLRP3 assembly and activation(Tapia-Abellan et al., 2021). TWIK2-mediated K^+^ efflux thus serves as a checkpoint for the initiation of trained immunity as well as maladaptive inflammatory signaling mediated by NLRP3(Di et al., 2018). This function of TWIK2 in initiating AMφ trained immunity remains uncertain.

Our previous work outlined a mechanism wherein extracellular ATP induces Rab-11-mediated outward translocation of the TWIK2 K^+^ efflux channel(Huang et al., 2023). Membrane-associated TWIK2 was sufficient to drive NLRP3-activating potassium efflux, significantly enhancing inflammatory innate immune function(Di et al., 2018; Huang et al., 2023). Here, we show that TWIK2, after PM insertion (induced by ATP), is re-internalized into AMφ upon infection while remaining associated with the phagosome membrane. TWIK2 is localized in the phagosome membrane and increases phagosome ionic strength by transporting K^+^ from the cytosol into the phagosome, which favors phagosomal protease activation, thus enhancing bactericidal activity. Therefore, TWIK2 functions both in the PM and phagosomes to optimize bacterial killing, and the channel can be modulated to promote AMφ training. The regulatable association of TWIK2 with the PM and phagosomes triggered by ATP mediates trained immunity in AMφ yet avoids excessive inflammation.

## Results

### TWIK2 is required for ATP induced AMφ training

**Figure 1.**
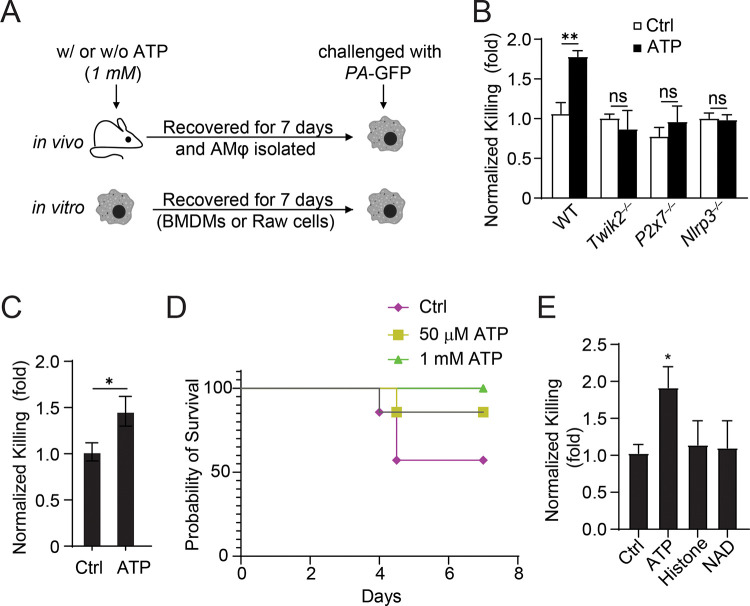
Central role of TWIK2 in triggering ATP-induced AMφ training of bacterial killing. **(A).** Diagram of the ATP-induced AMφ training model in vivo. WT or KO mice were exposed to ATP (1mM, i.n.) and AMφ were isolated on day 7 to determine acquisition of training. For in vitro training, BMDMs or RAW cells were trained with or without ATP (1mM) and recovered for 7 days. **(B).** AMφ subjected to training as described in A were incubated with live PA-GFP (MOI:50–100), PA killing was evaluated by decreased GFP fluorescence. **(C)**. BMDMs subjected to in vitro training (1mM ATP for 7 days as described in A) were incubated with live PA-GFP (MOI:50–100) and PA killing activity were determined. **(D).** Survival of control or ATP pretrained mice after receiving 40mL of 5*10^6^ c.f.u. of P. aeruginosa intranasally instilled, n = 5. **(E)** Comparison of bactericidal activity of AMφ trained with various DAMPs: ATP, histone or NAD. ATP showed the most dramatic response. ∗, p<0.05, ∗∗∗, p<0.001 (n = 3).

AMφ are exposed to inhaled particles and microbes, thus representing ideal cells to study trained immunity. Since ATP is widely appreciated as a ubiquitous DAMP(Coutinho-Silva and Savio, 2021) that can activate NLRP3 via P2X7-TWIK2 signaling in macrophages(Di et al., 2018; Huang et al., 2023), we investigated the role of ATP in the induction of immune memory. WT, *Twik2*^*−/−*^, *P2×7*^*−/−*^, and *Nlrp3*^*−/−*^ mice were intranasally exposed to ATP (1mM, i.n.), and AMφ were isolated on day 7 to determine the acquisition of training. The cells were further challenged with GFP-expressing *Pseudomonas aeruginosa* (GFP-*PA*, Fig. 1A). To assess the microbial killing activity, we measured the GFP expression levels of PA within AMφ in the presence or absence of extracellular ATP. AMφ from ATP-treated WT mice showed markedly augmented microbial killing as compared with controls (Fig. 1B). Similar results were observed in bone marrow-derived macrophages (BMDM) trained with ATP in vitro (Fig. 1C). Importantly, bactericidal activity remained unaffected in AMφ from TWIK2, P2X7 or NLRP3 null mice (Fig. 1B), indicating a crucial role of P2X7-TWIK2-NLRP3 signaling in ATP-mediated AMφ training. These findings support a model that ATP functions through the TWIK2 channel to activate training. Indeed, ATP-trained mice had a better survival rate after being infected intratracheally with PA, as compared with untrained controls (Fig. 1D). The induction of memory was not observed in AMφ treated with other DAMPs such as histone or NAD (Fig. 1E) highlighting the importance of ATP signaling.

### ATP induces endosomal TWIK2 PM translocation in Mφ.

**Figure 2.**
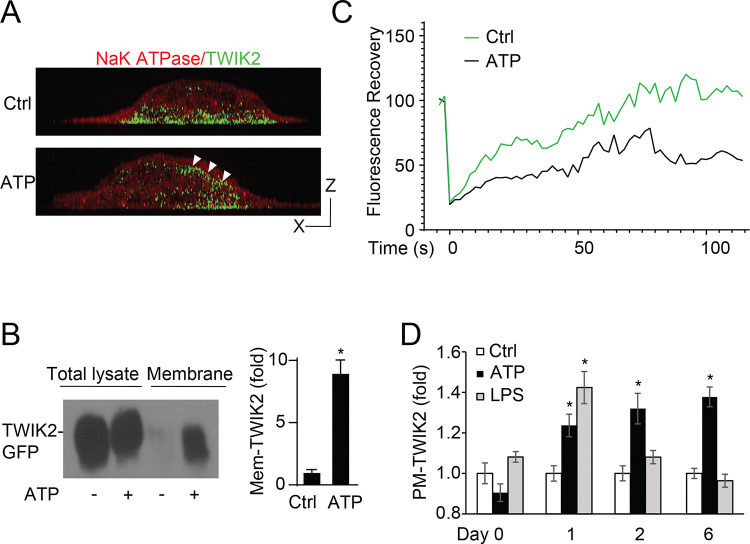
Assessment of TWIK2 PM and phagosome translocation. **A.** Images of RAW cells expressing TWIK2-GFP post-ATP challenge (1mM, PM stained in red). White arrowheads show PM translocated TWIK2. Bar, 5μm. **B.** Western blot analysis of ATP trained or untrained macrophage lysate after ultracentrifuge fractionation. Membrane and non-membranous fractions were compared to assess the relative abundance of TWIK2 and the Na-K ATPase with and without ATP training. **C.** Ratiometric fluorescence intensity analysis of TWIK2-GFP signal within 1 micrometer of the plasmalemma to the corresponding TWIk2-GFP cytoplasmic intensity, highlighting enhanced and long-term membrane association after ATP treatment. **D**. Duration of PM-TWIK2 untreated or ATP or LPS treated cells. *, P<0.05, **, P<0.01, ***, P<0.001, compared with control (n = 3).

We hypothesized that TWIK2 translocation to the plasma membrane (PM) and phagosome determines its role in macrophage training. Therefore, we studied TWIK2 dynamics by imaging TWIK2 PM translocation using TWIK2-GFP. We observed in RAW264.7 cells that TWIK2 translocated to the PM within 2 min post-ATP (Fig. 2A); this observation was confirmed by membrane fractioned TWIK2 (Fig. 2B). After ATP training, membrane-bound TWIK2 demonstrated less diffusion as measured by fluorescence recovery after photobleaching (FRAP) (Fig. 2C). Surprisingly, PM TWIK2 could be positioned at the PM for as long as 6 days following ATP activation (Fig. 2D). These data suggest PM association may sustain functionally relevant AMφ training. Interestingly, LPS itself showed no such effect on TWIK2 PM association (Fig. 2D) suggesting the importance of ATP signaling in activating the process.

**Figure 3.**
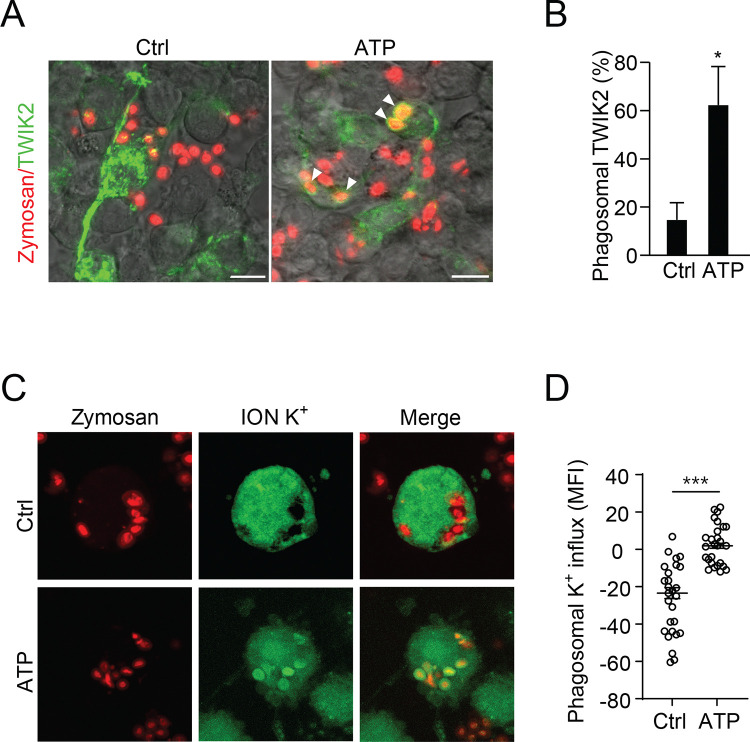
PM-TWIK2 re-internalizes into phagosomes upon phagocytosis. **A.** Fluorescence images of TWIK2-GFP expressing RAW 264.7 cells phagocytosing zymosan (red) trained with or without ATP. **B.** Quantification of A. We observed significantly greater phagosome-associated TWIK2 in ATP-trained cells vs. controls. Arrowheads point to phagosomes with TWIK2-GFP. ∗, p<0.01 compared with untreated control (n = 3). **C, D**, Representative images (C) and quantification (D) of K^+^ enrichment (indicated by a specific K^+^ dye, ION K^+^, green) in phagosomes of control and ATP-trained BMDMs, ∗∗∗, p < 0.001.

Upon bacterial phagocytosis, TWIK2 translocation to the phagosomal membrane may promote enhanced bactericidal activity by regulating phagosome acidification and ionic strength(Reeves et al., 2002). Therefore, we compared phagocytosis in TWIK2-GFP expressing RAW264.7 cells trained with ATP vs. control cells. While both groups (ATP-trained and untrained) efficiently endocytosed zymosan, ATP training markedly increased TWIK2-GFP phagosomal membrane translocation as compared to untrained control cells (Fig. 3A, B). Thus, TWIK2, after its PM insertion, is internalized in its proper topology in the membrane of bacteria-laden phagosomes.

**Figure 4.**
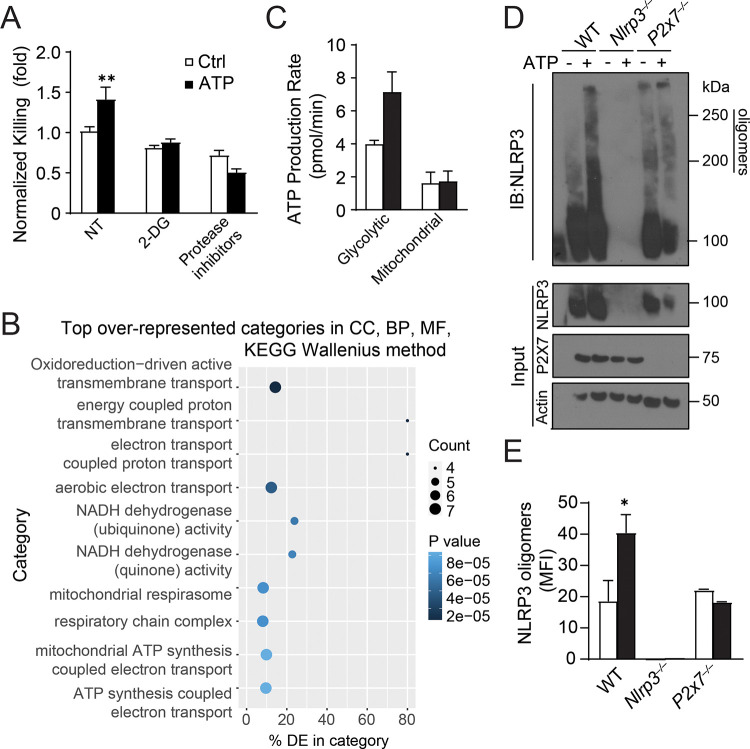
ATP training regulates transcriptional profile and metabolic reprogramming of Mφ. **A.** Normalized PA killing ability of BMDMs trained with or without ATP in the presence of metabolic inhibitors 2DG or protease inhibitors. **B.** RNA isolated from BMDMs trained with or without ATP was mapped to the *Mus musculus* genome and subsequently sorted into GO terms in Galaxy. Shown are the top 10 GO categories overrepresented in ATP trained BMDMs. **C.** Basal glycolytic and mitochondrial metabolism in BMDMs trained with or without ATP as exhibited by Seahorse assay. **D, E.** Western blot (D) and quantification (E) of NLRP3 assembly in BMDMs trained with or without 1mM ATP and challenged with 1.0 MoI PA for 2 hours. These cells were lysed with DSP crosslinker and assessed for NLRP3 oligomerization, n=3.

Next, we compared phagosomal K^+^ changes after bone marrow-derived macrophages (BMDM) phagocytosed zymosan. We found that untrained cells exhibited little if any, phagosome K+ influx, whereas ATP-trained BMDM markedly increased phagosome K^+^ influx (indicated by a specific K^+^ dye, ION K^+^, green) (Fig. 3C, D). Thus, TWIK2 translocates to the phagosome membrane upon phagocytosis of trained macrophages to increase the phagosome ionic strength, which favors bacterial clearance. K^+^ influx in phagosomes via TWIK2 renders phagosomes hypertonic, which is known to activate hydrolytic proteases through the fusion of granules(Vieira et al., 2002). Indeed, ATP-induced AMφ training effects, as measured by augmented bactericidal activity, were diminished in AMφ treated with protease inhibitors (Fig. 4A).

### ATP-TWIK2 signaling regulates transcriptional profile and metabolic reprogramming of AMφ

Since epigenetic remodeling and metabolic reprogramming are two processes that may be fundamental to the mechanisms of trained immunity(Netea and Joosten, 2018), we further examined how the ATP training regulates transcriptional profile in macrophages. To identify transcriptional changes of ATP-exposed and control cells, BMDMs were trained with or without ATP for 7 days. GO analysis of RNA-seq has been performed in trained and control cells. We discovered that the top markedly upregulated genes after ATP training are metabolic genes (Fig. 4B). To further assess the effect of the differential metabolic programs of trained and untrained macrophages, we evaluated each set via the SEAHORSE ATP production rate assay. This revealed that ATP-treated BMDMs displayed increased metabolic activity, specifically by way of glycolytic ATP production (Fig. 4C). Importantly, ATP-induced augmented bactericidal activity was abolished in macrophages treated with metabolic inhibitor 2-deoxy-d-glucose (2-DG) (Fig. 4A).

Due to the dependence of bacteria-killing on NLRP3 inflammasome, we assessed the role of ATP training on the NLRP3 inflammasome activation. By lysing cells in the presence of cross-linker, we assessed the extent of high molecular weight NLRP3 complex formation in BMDMs with two hours of bacteria co-incubation. In untrained macrophages, exposure to bacteria resulted in the formation of 200 and 250 kDa complexes (Fig. 4D, E), indicating NLRP3 inflammasome assembly. This oligomerization was enhanced in ATP trained BMDMs, but was absent in P2X7 knockout macrophages, in which NLRP3 oligomerization exhibited no difference between trained or nontrained P2X7 null BMDMs (Fig 4D, E). NLRP3 is known to interact with some metabolic enzymes such as 6-phosphofructo-2-kinase/fructose-2,6-bisphosphatase 3 (PFKFB3)(Finucane et al., 2019) and phosphoglycerate kinase 1 (PGK1)(Zhu et al., 2024). Hence, ATP-mediated macrophage training may enhance metabolic activity through the NLRP3 inflammasome.

### ATP training rescues pneumonia-induced immunosuppression.

**Figure 5.**
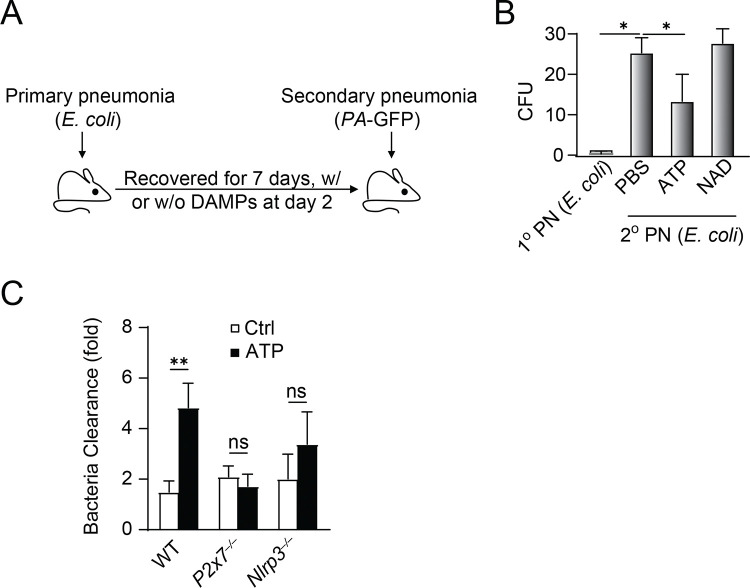
ATP training rescues lung immunosuppression caused by pneumonia. **A.** Diagram of the double-infection model of primary pneumonia (1° PN) with 1×10^6^ *E. coli* and secondary pneumonia (2° PN) with 1×10^6^ GFP-*PA*. **B.** Colony-forming units (CFU) per ml of BAL with 1° or 2° pneumonia (both induced by *E. coli*). ∗, p < 0.05, n = 3. **C.** Bacterial killing ability in WT or designated KO mice trained with or without ATP in the double exposure model.

Bacterial pneumonia after influenza is a leading cause of severe respiratory infections worldwide(Metzger and Sun, 2013). Unlike adenoviral infection(Yao et al., 2018), influenza infection abrogates AMφ-dependent bacteria clearance(Verma et al., 2020), indicating that context (type, dose and duration of infection) may be critical for AMφ phenotypes. We utilized a double-infection model to mimic the clinical scenario. Mice were first subjected to bacterial (*E. coli* or *S. aureus*) pneumonia, left to recover with or without ATP training (50 mM, i.t) for 7 days, and then infected i.t. with YFP *PA* to cause secondary pneumonia (Fig. 5A). We found that the bacterial burden in mice doubly infected with *E. coli* was greater than in mice with primary infection but was markedly reduced with ATP treatment (Fig. 5B), which was not observed in other DAMP treatment such as NAD (Fig. 5B). This rescue effect by ATP was not observed in P2X7 and NLRP3 KO mice, indicate the importance of P2X7-NLRP3 singling in ATP mediated macrophage training (Fig. 5C). These results demonstrate that AMφ develop with impaired capacity to ingest or kill bacteria during pneumonia and the dysfunction can be rescued by ATP training.

## Discussion

Lungs are continuously exposed to environmental pathogens and require a rapid immune response to ensure host survival. AMφ are key cells of this innate defense, and induction of trained innate immunity in these cells enhances their capability. Leveraging the trained immunity of AMφ would be beneficial to promote host defense function while preventing auto-inflammatory injury, the key feature of ALI/ARDS. we discovered that ATP could train AMφ through the ATP-P2X7-TWIK2 pathway described above. Our data suggest that during homeostasis, TWIK2 resides in the endosomal compartment. In response to increased extracellular ATP, endosomal TWIK2 is translocated to the PM and remains localized in the phagosome membrane upon phagocytosis of bacteria. This raises the intriguing concept of augmenting the activity of TWIK2 that will train AMφ in multiple ways. We posit that a noteworthy element of this function is the tunability of TWIK2 association with PM and in phagosomes and hence the ability of TWIK2 to control innate immunity (the definition of training). We hypothesize that tunability of AMφ is a central function of these cells and it requires upregulation and/or duration of ATP-mediated TWIK2 PM translocation. Therefore, controlling the magnitude and duration of TWIK2-mediated NLRP3 inflammasome activation provides potential therapeutic targets for the treatment of ALI.

Our data showed other DAMPs such as histone or NAD did not elicit trained immunity of Mφ indicating the importance of ATP signaling. Different pathogens or their products exploit distinct mechanisms to train AMφ. For example, adenoviral infection induced AMφ trained immunity requires IFN-γ produced by T-cells(Yao et al., 2018), while LPS-induced AMφ training depends on type 1 interferon, IFN-β(Zahalka et al., 2022). LPS trained AMφ also exhibited upregulated expression of the efferocytosis receptor MERTK and greater capacity for efferocytosis of cellular debris(Chakraborty et al., 2023). Thus, generalizations may not be possible. In addition, since viral, LPS or bacterial challenge can generate ATP, it will be interesting to test whether ATP-TWIK2 represents a common mechanism which applies to different means of training.

There is the possibility that training and suppression are both induced upon different pathogen challenges(Verma et al., 2020; Yao et al., 2018). Both the nature of the pathogen and the pathogen dose play critical roles in determining these opposing effects(Bauer et al., 2018). For example, low-dose LPS promotes hyperactive responses while high-dose LPS prolongs inhibition in AMφ(Bauer et al., 2018; Fu et al., 2012; Zahalka et al., 2022). In addition, trained innate immunity is different from a primed immune response. In contrast to priming, trained immunity returns to the basal level following removal of the first stimulus(Divangahi et al., 2021). However, in response to secondary challenges, both gene transcription and cell functions are enhanced at much higher levels than those in the primary challenge(Divangahi et al., 2021). In our training model, the functional program of AMφ returns to basal level after first stimulation. The training model is fundamentally different from models of priming in which the stimulus is either maintained for a long period of time to induce priming or secondary stimulation is performed very quickly after the initial priming.

The discovery of trained immunity has profound implications for both immunity to infectious diseases and the development of novel therapeutic strategies. Despite the benefits, there are concerns about the potential harmful effects of a chronically trained, or partially activated, immune system(Netea et al., 2016). Thus, Understanding the mechanisms that govern the induction, persistence, and resolution of trained immunity is essential for developing strategies to harness its benefits while minimizing its risks. Our findings reveal that regulatable association of TWIK2 with the PM and phagosomes triggered by ATP mediates trained immunity in AMφ. The tunability of AMφ is a central function of these cells and it requires upregulation and/or duration of ATP-mediated TWIK2 PM translocation, which will lead to understanding new immunotherapeutic approaches.

## Methods

### Cell Culture

RAW 264.7 cells were cultured in Dulbecco’s Modified Eagle Medium (DMEM) supplemented with 10% fetal bovine serum (FBS) and 1% penicillin-streptomycin. Cells were maintained at 37°C in a humidified atmosphere with 5% CO_2_. Bone marrow-derived macrophages (BMDMs) were isolated from the femurs and tibias of C57B/L6J or C57B/L6J -derived mice (8–16 weeks old, males and females harvested approximately equally) and differentiated using 0.22 μm filtered 10% L929-conditioned media. Cells were cultured in RPMI 1640 with 10% FBS and 1% penicillin-streptomycin.

### Membrane Isolation

Plasma membranes were isolated using the Subcellular Protein Fractionation Kit for Cultured Cells (Thermo Fisher Scientific 78840) according to the manufacturer’s instructions. Fractionation was verified as described by Thermo Scientific for the Sorvall MTX Micro-Ultracentrifuge and S55-A2 Rotor, modified from Smart et al 1995. Briefly, Post-nuclear lysate of ATP-trained and untrained cells was layered on a 0/30% isotonic percoll stepwise gradient and separated via ultracentrifugation at 84000 × g for 30 minutes at 4°C, the membrane-containing lysate was collected from the interface, diluted in ice-cold PBS, and further ultracentrifuged at 105,000 × g for 90 minutes at 4°C to clear percoll and obtain purified membranes.

### Microscopy

Structured illumination microscopy (SIM) was performed using an DeltaVision OMX 3D-SIM microscope (GE Healthcare) with 3D-SIM reconstruction software, using a 60x, 1.42 NA Oil PSF Objective (Olympus PlanApo N). Membrane GFP intensity was also measured using a Leica LSM880 confocal microscope *objective* and analyzed with ImageJ software version 1.54g.

### ATP-mediated training

For ATP training in cell culture, ATP salt (Thermofisher L14522.06) was dissolved to 1.0 M in cell culture-grade water. This stock was diluted to a final concentration of 1mM in complete RPMI media and subsequently applied to cells. Media was changed after 24 hours, and cells were maintained for an additional 6 days until challenge or harvest. Bacterial cultures for challenge (E. coli strain DH5a and/or GFP-transformed P. aeruginosa strain PA-01) were grown in Luria Broth or Luria Broth plus 0.1 mg/ml Ampicillin, respectively, for 12–16 hours. Culture density was determined by empirically-determined OD600 and bacteria (or AF594-tagged zymosan beads (Thermofisher Z23374)) were diluted to 2.5×107 c.f.u (or beads) per mL and co-cultured with cells in culture conditions described above. Primary challenge (E. coli) was co-cultured in complete RPMI 1640 media for 24 hours before washing with PBS and replenishing sterile complete media. P. aeruginosa and Zymosan bead endpoint challenge was co-cultured in sterile PBS for 2 hours before final assessment. Metabolism inhibitors 2-Deoxy-D-glucose, NG52, and PFK158 (All from SelleckChem) were applied to cells in incomplete media (not supplemented with FBS or P/S) at final concentrations of 1mM three hours prior to insult, 10μM one hour prior to insult, and 15 μM one hour prior to insult, respectively. P. aeruginosa was then applied directly to culture to assess bacterial clearance as described below.

ION Potassium Green (5 μM in DMSO; Abcam AB142806) BCECF-AM (3 μM in DMSO; Abcam AB143463), or similar volume DMSO vehicles were applied to cells concurrently with terminal challenge, and assessed by confocal microscopy as described above, or by CytoflexS Flow cytometry.

### Fluorescence Recovery After Photobleaching (FRAP)

FRAP was performed to assess the dynamics of TWIK2-GFP on phagosomal membranes. Cells were bleached using a 488 nm laser at maximum laser power, and recovery was monitored for 2 minutes with images captured every 2 seconds, using an *objective *. Analysis was conducted using ImageJ software version 1.54g, and percent recovery was determined by ratiometrically comparing each measurement to the average of both pre-bleach measurements.

### Bacterial Killing Assay

An in vitro bacterial killing assay was conducted using P. aeruginosa (strain PA-01 GFP). Cells were infected at a multiplicity of infection (MOI) of 1.0. After 2 hours of incubation, cells were harvested and serial dilutions were plated on LB agar with 0.1mg/ml Ampicillin to quantify remaining bacteria. Bacterial colonies were counted after overnight incubation at 37°C. Colony numbers were normalized to ‘fold killing’ by dividing the average of all negative control samples by that of each experimental group to provide ratiometric comparison.

### Seahorse ATP Production Assay

ATP production was quantified using a Seahorse XF Analyzer (Agilent Technologies). BMDM progenitors were seeded at 100,000 cells/well and differentiated in Seahorse XF96 cell culture microplates. Rotenone/Antimycin and Oligomycin A were loaded, and basal and maximal respiration rates were measured, according to the manufacturer’s protocol.

#### Statistical analysis.

All experiments were performed at least three times. Representative data are shown in the paper. An independent experiments two-tailed Student’s t-test was used as the statistical assay for comparisons. Significant differences between samples were indicated by P < 0.05.
